# Improved Data-Driven Collective Variables for Biased Sampling through Iteration on Biased Data

**DOI:** 10.1021/acs.jpcb.5c02164

**Published:** 2025-06-13

**Authors:** Subarna Sasmal, Martin McCullagh, Glen M. Hocky

**Affiliations:** † Department of Chemistry and Simons Center for Computational Physical Chemistry, New York University, New York, New York 10003, United States; ‡ Department of Chemistry, Oklahoma State University, Stillwater, Oklahoma 74078, United States

## Abstract

Our ability to efficiently sample conformational transitions between two known states of a biomolecule using collective variable (CV)-based sampling depends strongly on the choice of the CV. We previously reported a data-driven approach to clustering biomolecular configurations with a probabilistic clustering model termed shapeGMM. ShapeGMM is a Gaussian mixture model in Cartesian coordinates, with means and covariances in each cluster representing the harmonic approximation to the conformational ensemble around a metastable state. We subsequently showed that linear discriminant analysis on positions (posLDA) produces good reaction coordinates to characterize the transition between two of these states, and moreover, they can be biased to produce transitions between the states using metadynamics-like approaches. However, the quality of these posLDA coordinates depends on the amount of data used to characterize the states, and here, we demonstrate the ability to systematically improve them using enhanced sampling data. Specifically, we demonstrate that improved CVs for sampling can be generated by iteratively performing biased sampling along a posLDA coordinate and then generating a new shapeGMM model from biased data from the previous iteration. The new coordinates derived from our iterative approach show a substantial improvement in being able to induce transitions between metastable states and to converge a free energy surface.

## Graphical abstract

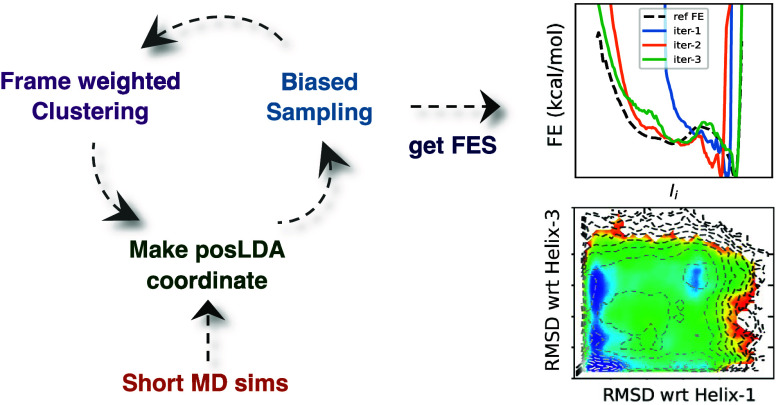

## 1 Introduction

Molecular dynamics (MD) is a powerful approach for studying complex biochemical processes.^1^ However, many critical events, such as protein folding and allosteric regulation of enzymes, occur on time scales that are often inaccessible to conventional MD due to the so-called rare event problem.^1,2^ In these cases, the system becomes trapped in an initial metastable state, unable to overcome high free energy barriers that separate different regions of the free energy landscape. This limitation is particularly pronounced in large systems with many degrees of freedom, where fully sampling all relevant states is nearly impossible, even with very long MD simulations.

Over the years, numerous enhanced sampling techniques have been developed to alleviate this challenge by facilitating more frequent transitions between different states of a system.^3,4^ One prominent class of these methods relies on collective variables (CVs), where an external bias is applied as a function of carefully chosen CVs. An ideal CV is thought to capture the slowest modes of motion that are responsible for significant conformational changes in macromolecules. By applying a bias to enhance fluctuations in one or several CVs, these methods encourage the system to explore low-probability regions of the free energy surface. However, the effectiveness of CV-based enhanced sampling techniques depends heavily on the choice of CVs, which can be particularly challenging for complex systems.

There are numerous methods designed to identify “optimal” CVs for a given system, each with its own strengths and limitations. Some approaches employ simple linear dimensionality reduction techniques, while others leverage machine learning (ML) and deep learning algorithms to construct sophisticated nonlinear coordinates.^5–12^ Interestingly, most of these methods rely on training with initial, under-sampled MD simulation data, which often lacks sufficient information about different metastable states and their transitions. The effectiveness of these approaches is inherently dependent on the quality of the sampling used for training. The resulting CV, obtained from this limited dataset, serves as a fixed reaction coordinate that is subsequently biased in enhanced sampling simulations to achieve a well-converged free energy surface (FES).

Recent studies have introduced a different strategy for identifying the optimal reaction coordinates. These approaches employ an iterative scheme that refines the initially defined CV by leveraging reweighted data from successive biased simulations.^13–22^ Unlike traditional methods, where the CV remains fixed, this iterative process continuously improves the coordinate as it is trained on progressively better-sampled data from each iteration of the biased simulations. This CV refinement enhances the accuracy and efficiency of sampling relevant configurations, leading to a more reliable exploration of the free energy landscape. While these methods are highly efficient, they are computationally expensive. The resulting coordinates often lack clear physical interpretability and are sensitive to hyperparameters and neural network architecture, requiring careful tuning for optimal performance.

Here, we present an iterative scheme to improve our previously reported position linear discriminant analysis (posLDA) approach.^9^ Linear discriminant analysis produces the linear coordinate that optimally separates two labeled sets of data, and posLDA applies this approach to the Cartesian positions of biomolecules (which requires additional translation and rotation steps below). posLDA offers the advantage of being potentially interpretable, since the coefficients of the linear coordinate correspond to a motion of the molecule in Cartesian space. Moreover, in our previous study, we showed that it can serve as a “good” reaction coordinate between two states, in the sense that we found it was correlated with the “committor” (likelihood of transitioning to either of the two basins), with a committor value of ∼0.5 at the putative transition state identified by posLDA.^9^ In this study, we aim to enhance posLDA coordinates through an iterative refinement process, since as a data-driven CV, the initial trained coordinate is inherently limited by the extent of sampling available for the two reference states a priori.

Our iterative process starts by creating an initial CV using data from short, unbiased MD simulations. Enhanced sampling is then performed along this coordinate, and the free energy surface is assessed for convergence. If convergence is not achieved, biased samples are reweighted and clustered using our frame-weighted shapeGMM^23^ method to further refine the coordinate. This process repeats until the free energy surface or another relevant observable converges, providing an optimal reaction coordinate for efficient sampling. We have applied this protocol on two systems of increasing complexity: a nine-residue peptide (Aib)_9_ and a 35-amino acid fast-folding Nle/Nle mutant Villin headpiece (also known as HP35). In both cases, iteration improves both the stability (meaning more aggressive biasing parameters can be used) and the ability to converge a FES when biasing along that CV. This approach is implemented in tools that we have made available within an updated shapeGMMTorch python package^24^ with biased simulations available in a number of MD simulation packages via our sizeshape PLUMED module.^9,25^

## 2 Theory and Methods

### 2.1 Iteration Process

The iterative scheme employed in this work combines our previous three procedures^9,23,26^ in a straightforward yet effective approach, as illustrated by the flowchart in Figure 1. The focus of our work is on identifying a reaction coordinate that connects two specific states of a given system.

**Figure 1:**
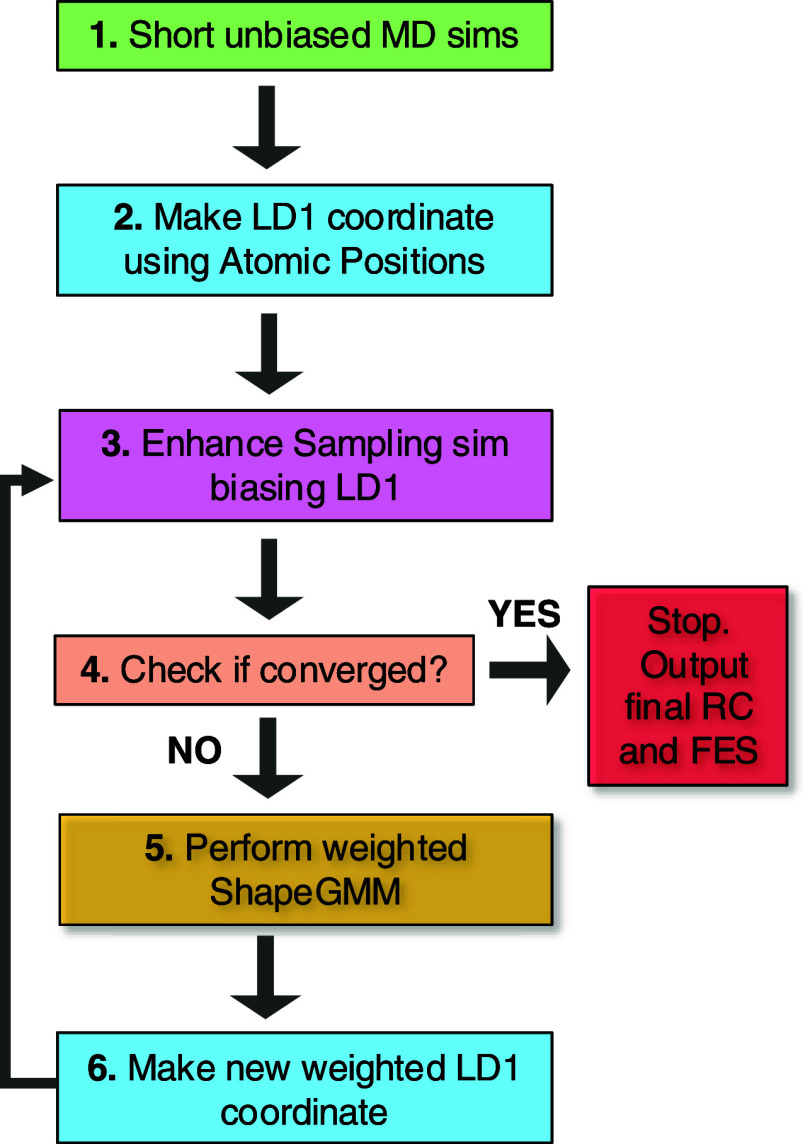
Workflow for the iteration scheme. There are 6 steps: 1. short unbiased MD simulations are performed starting from two states of interest; 2. use the LDA method to make LD1 coordinate; 3. perform enhanced sampling simulation, biasing LD1 coordinate; 4. check the convergence of FES within a given threshold; if not converged, 5. apply frame-weighted shapeGMM on biased data to identify new states; 6. apply frame-weighted LDA between two newly identified states to generate a new weighted LD1 coordinate. Repeat steps 3 to 6 until the simulation converges.

The process begins with two short unbiased MD simulations initiated from each state (data from a long unbiased MD trajectory can be used if both states of interest are sufficiently well represented). From the resulting labeled MD simulation data, an initial linear discriminant analysis (LDA) coordinate is constructed. In the next step, an enhanced sampling technique such as metadynamics (MetaD) or the on-the-fly probability enhanced sampling variant (OPES-MetaD)^27,28^ is used, applying a bias along the LDA coordinate within an MD simulation (see Section 2.4). Following this, the convergence of the free energy (FE) is assessed. If the FE surface has not yet converged, then the workflow proceeds to the next stage, where the frame-weighted shapeGMM method is used to cluster the biased samples. This method accounts for the nonuniform weights associated with each biased sample, effectively reweighting them to provide an unbiased estimate of the clusters. Subsequently, we compute the Bhattacharyya distances between the new clusters and the initially defined reference states, selecting the clusters with the smallest distances. A new weighted LDA coordinate is then computed using reweighted samples from these clusters. The process iterates through steps 3 to 6 until the free energy surface (FES) or another relevant physical observable converges within a predefined threshold. The reaction coordinate obtained at the end of this iterative process serves as an optimized bias coordinate, enhancing the efficiency of FES sampling when employed in enhanced sampling simulations. In practice, it may be difficult to assess the convergence of the FES given the finite time of sampling available (step 4), and so here we also highlight the efficiency of exploration, namely, how frequently the biased CV and other physically intuitive CVs of interest transit between the values for the two target states, which may be a useful proxy in the case of an exploration method such as MetaD.

### 2.2 Weighted ShapeGMM

In shapeGMM, a particular configuration of a macromolecule is represented by a particle position matrix, ***x***_*i*_, of order *N* × 3, where *N* is the number of particles being considered for clustering. To account for translational and rotational invariance, the proper feature for clustering purposes is an equivalence class,

1 $$\left[x_{i}\right]=\left\{x_{i}R_{i}+1_{N}{\mathrm{\xi ⃗}}_{i}^{T}:{\mathrm{\xi ⃗}}_{i}\in R^{3},R_{i}\in \mathrm{SO}\left(3\right)\right\}$$

where ${\mathrm{\xi ⃗}}_{i}$ is a translation in $R^{3}$ , ***R***_*i*_ is a rotation $R^{3}\to R^{3}$ , and **1**_*N*_ is the *N* × 1 vector of ones. [***x***_*i*_] is thus the set of all rigid body transformations, or the orbit, of ***x***_*i*_.

The shapeGMM probability density is a Gaussian mixture given by

2 $$P\left(x_{i}\right)=\overset{K}{\underset{j=1}{\sum }}\phi_{j}N\left(x_{i}|\mu_{j},\Sigma_{j}\right)$$

where the sum is over the *K* Gaussian mixture components, ϕ_*j*_ is the weight of component *j*, and *N*(***x***_*i*_ | **μ**_*j*_, **Σ**_*j*_) is a normalized multivariate Gaussian given by

3 $$N\left(x_{i}|\mu ,\Sigma \right)=\frac{\exp\left[-\frac{1}{2}{\left(g_{i}^{-1}x_{i}-\mu \right)}^{T}\Sigma^{-1}\left(g_{i}^{-1}x_{i}-\mu \right)\right]}{\sqrt{{\left(2\pi \right)}^{\left(3N\right)}\det\Sigma }}$$

where **μ** is the mean structure, **Σ** is the covariance, and $g_{i}^{-1}x_{i}$ is the element of the equivalence class [***x***_*i*_] that minimizes the squared Mahalanobis distance in the argument of the exponent.

Determining the proper transformation, *g*_*i*_, is achieved by translating all frames to the origin and then computing an optimal rotation matrix. Cartesian and quaternion-based algorithms for determining optimal rotation matrices are known to exist for two forms of the covariance: **Σ** ∝ ***I***_3*N*_ ^29,30^ or **Σ** = **Σ**_*N*_ ⊗ ***I***_3_,^31,32^ where **Σ**_*N*_ is the *N* × *N* covariance matrix and ⊗ denotes a Kronecker product. For a comparison of clustering with these two covariance types, see the original shapeGMM paper.^26^ In this paper, we employ only the more general Kronecker product covariance because of its improved ability to separate states with heterogeneous covariance.

While using input data from an enhanced sampling simulation, we take nonuniform frame weights into account by performing weighted averages in the expectation maximization estimate of model parameters $\left\{{\mathrm{\phi ̂}}_{j},{\mathrm{\mu ̂}}_{j},{\mathrm{\Sigma ̂}}_{j}\right\}$ . Considering a normalized set of frame weights, {*w*_*i*_} where $\overset{M}{\underset{i=1}{\sum }}w_{i}=1$ for *M* frames, their contribution to the probability can be accounted for by weighting the estimate of the posterior distribution of latent variables, *Z*_*i*_ ∈ {1 ,..., *K*}, for each mixture component, *j*:

4 $$\gamma_{Z_{i}}\left(j\right)=w_{i}\frac{{\mathrm{\phi ̂}}_{j}N\left(x_{i}|{\mathrm{\mu ̂}}_{j},{\mathrm{\Sigma ̂}}_{j}\right)}{\overset{K}{\underset{j=1}{\sum }}{\mathrm{\phi ̂}}_{i}N\left(x_{i}|{\mathrm{\mu ̂}}_{j},{\mathrm{\Sigma ̂}}_{j}\right)}$$

The frame weight will propagate to the estimate of component weights, means, and covariances in the maximization step through $\gamma_{Z_{i}}\left(j\right)$ :

5 $${\mathrm{\phi ̂}}_{j}=\overset{M}{\underset{i=1}{\sum }}\gamma_{Z_{i}}\left(j\right)$$

6 $${\mathrm{\mu ̂}}_{j}=\frac{\overset{M}{\underset{i=1}{\sum }}\gamma_{Z_{i}}\left(j\right)g_{i,j}^{-1}x_{i}}{\overset{M}{\underset{i=1}{\sum }}\gamma_{Z_{i}}\left(j\right)}$$

7 $${\mathrm{\Sigma ̂}}_{j}=\frac{\overset{M}{\underset{i=1}{\sum }}\gamma_{Z_{i}}\left(j\right)\langle {\mathrm{\Sigma ̂}}_{N}\rangle_{i}}{\overset{M}{\underset{i=1}{\sum }}\gamma_{Z_{i}}\left(j\right)}⊗I_{3}$$

Additionally, the log likelihood per frame is computed as a weighted average

8 $$\ln\left(L\right)=\overset{M}{\underset{i=1}{\sum }}w_{i}\ln\left(\overset{K}{\underset{j=1}{\sum }}{\mathrm{\phi ̂}}_{j}N\left(x_{i}|{\mathrm{\mu ̂}}_{j},{\mathrm{\Sigma ̂}}_{j}\right)\right)$$

### 2.3 Frame-Weighted LDA (wLDA)

LDA is a supervised classification technique that projects data into a lower-dimensional space.^33^ The lower-dimensional space is determined by simultaneously maximizing the projection of the between-class scatter matrix and minimizing the projection of the within-class scatter matrix. In our prior work, we demonstrated that the application of LDA on aligned particle positions produces a good one-dimensional reaction coordinate that best separates two states.^9^ In the frame-weighted LDA approach, one can use input data obtained from enhanced sampling by incorporating nonuniform weights of the samples to account for the relative probabilities of different classes. To do so, we include weights corresponding to each configuration in the estimation of the scatter matrices. To estimate the scatter matrices, we first perform a frame-weighted Kronecker covariance-based alignment of frames associated with the chosen states. The scatter matrices are then estimated directly from this data set.

For *K* different clusters, this is achieved by first computing the weighted within-cluster scatter matrix,

9 $$S_{W}^{w}=\overset{K}{\underset{j=1}{\sum }}\underset{i\in N_{j}}{\sum }w_{i}\left(x_{i}-\mu_{j}\right){\left(x_{i}-\mu_{j}\right)}^{T}$$

and the between-cluster scatter matrix,

10 $$S_{B}^{w}=\overset{K}{\underset{j=1}{\sum }}W_{j}\left(\mu_{j}-\mu \right){\left(\mu_{j}-\mu \right)}^{T}$$

where $\mu_{j}=\frac{\underset{i\in N_{j}}{\sum }w_{i}x_{i}}{\underset{i\in N_{j}}{\sum }w_{i}}$ is the weighted mean of cluster *j*, {*w*_*i*_} are the normalized weights of individual samples such that $\overset{M}{\underset{i=1}{\sum }}w_{i}=1$ , and *N*_*j*_ is the set of samples that belong to cluster *j*. $\mu =\frac{\overset{K}{\underset{j=1}{\sum }}\underset{i\in N_{j}}{\sum }w_{i}x_{i}}{\overset{K}{\underset{j=1}{\sum }}\underset{i\in N_{j}}{\sum }w_{i}}$ is the overall weighted global mean, and $W_{j}=\underset{i\in N_{j}}{\sum }w_{i}$ is the total weight of samples in cluster *j*. The simultaneous minimization of within-cluster scatter and maximization of between-cluster scatter can be achieved by finding the transformation *G* that maximizes the quantity

11 $$\mathrm{Tr}\left({\left(G^{T}S_{W}^{w}G\right)}^{-1}G^{T}S_{B}^{w}G\right)$$

This maximization can be achieved through an eigenvalue/eigenvector decomposition, but such a procedure is only applicable when $S_{W}^{w}$ is nonsingular. The LDA method was reformulated in terms of the generalized singular value decomposition (SVD)^34^ extending the applicability of the method to singular $S_{W}^{w}$ matrices such as those encountered when using particle positions.

The result of a wLDA procedure is a matrix *G* with *K* – 1 nonsingular vectors that separate the *K* states. In principle, all of the *K* – 1 coordinates could be used to sample and monitor reaction, but the behavior of *K* > 2 has not been tested. Here, and in our previous work,^9^ we restrict the application to two labeled states such that wLDA will produce a vector, ***v***, of coefficients that best separate the two states.

We have implemented this modified approach in a weighted LDA Python package.^35^ While the wLDA approach is general, it is important to recognize that to use it in our position-LDA approach, each frame must be aligned in the same way as the training data before computing the dot product, as described in the next section. This procedure is implemented such that the coefficients can be used directly for sampling within the PLUMED sizeshape module.

### 2.4 Enhanced Sampling with LDA Coordinates

The LDA coordinate used here is a dot product of the vector ***v*** with the atomic coordinates ***x*** – **μ**, and it is given by^9^

12 l(x)=v·(R·(x(t)−ξ⃗(t))−μ)

To compute the value of the LDA coordinate *l* on the fly, we first translate ***x*** (***t***) by $\mathrm{\xi ⃗}\left(t\right)=\frac{1}{N}\overset{N}{\underset{i=1}{\sum }}{\mathrm{x⃗}}_{i}\left(t\right)-\frac{1}{N}\overset{N}{\underset{i=1}{\sum }}{\mathrm{\mu ⃗}}_{i}\left(t\right)$ , the difference in the geometric mean of the current frame and that of the reference configuration. Then, we compute ***R*** (*t*), the rotation matrix which minimizes the Mahalanobis difference between x(t)−ξ⃗ and **μ** considering a given **Σ**, as described in ref 26.

Enhanced sampling simulations on LDA coordinates were performed using well-tempered metadynamics (WT-MetaD) and on-the-fly probability enhanced sampling-metadynamics (OPES-MetaD), as implemented in PLUMED.^25,28,36,37^

WT-MetaD works by adding a bias formed from a history-dependent sum of progressively shrinking Gaussian hills.^27,38^ The bias at time *t* for CV value *s*_*i*_ is given by the expression

13 $$V\left(s_{i},t\right)=\underset{\tau <t}{\sum }he^{-V\left(s_{i},\tau \right)/\Delta T}e^{-s{\left(x\left(\tau \right)-s_{i}\right)}^{2}/{2\sigma }^{2}}$$

where *h* is the initial hill height, σ sets the width of the Gaussians, and Δ*T* is an effective sampling temperature for the CVs. Rather than setting Δ*T*, one typically chooses the bias factor γ = (*T* + Δ*T*)/*T*, which sets the smoothness of the sampled distribution.^27,38^ Asymptotically, a free energy surface (FES) can be estimated from the applied bias by $F\left(s\right)=-\frac{\gamma }{\gamma -1}V\left(s,t\to \infty \right)$ ^27,39^ or using a reweighting scheme.^27,40^

OPES-MetaD applies a bias that is based on a kernel density estimate of the probability distribution over the whole space, which is iteratively updated.^28,37^ The bias at time *t* for CV value *s*_*i*_ takes the form

14 $$V\left(s_{i}\right)=k_{B}T\left(\frac{\gamma -1}{\gamma }\right)\log\left(\frac{P_{t}\left(s_{i}\right)}{Z_{t}}+\epsilon \right)$$

Here in the prefactor, *T* is the temperature, *k*_B_ is Boltzmann’s constant, and γ is the bias factor. *P*_*t*_(*s*) is the current estimate of the probability distribution, and *Z*_*t*_ is a normalization factor that comes from integrating over sampled *s* space. Finally, $\epsilon =\exp\left(\frac{\Delta E}{k_{B}T}\frac{\gamma }{\gamma -1}\right)$ is a regularization constant that ensures the maximum bias that can be applied is Δ*E*.

As in WT-MetaD, *F*(*s*) can be directly estimated from *V*(*s*) by $F\left(s\right)\approx -\frac{\gamma }{\gamma -1}V\left(s\right)$ or through a reweighting scheme.^37^ We note that, as reported previously, standard OPES-MetaD focuses on converging as quickly as possible to an estimated FES rather than promoting further exploration.^41^ Details of the sampling parameters used for each system are given in Section 5.

## 3 Results and Discussion

### 3.1 Performing Iterations on (Aib)_9_

(Aib)_9_ is a nine-residue peptide formed from the achiral α-aminoisobutyryl, which exhibits two well-defined metastable states: left- and right-handed α-helices (see Figure 3). Due to the symmetry inherent in a helix made of achiral building blocks, both states must have equal statistical likelihood. In work by us and others,^9,15,42^ this symmetry was leveraged to benchmark sampling and clustering methods. Here, we applied the proposed iterative scheme to this system to test whether subsequent reaction coordinates generated via iteration allow for the efficient sampling of the underlying conformational ensemble.

The iterative process begins with an initial linear discriminant (LD1) coordinate derived from two short molecular dynamics (MD) simulations starting from the left- and right-handed states. This is followed by a WT-MetaD simulation biasing this coordinate (for details, see ref 9). The LD coordinate exhibited several transitions between extreme values representing the left- and right-helical states (Figure 2, top) within 500 ns. We subsequently performed three more iterations, with each WT-MetaD simulation also running for 500 ns (Figure 2).

**Figure 2:**
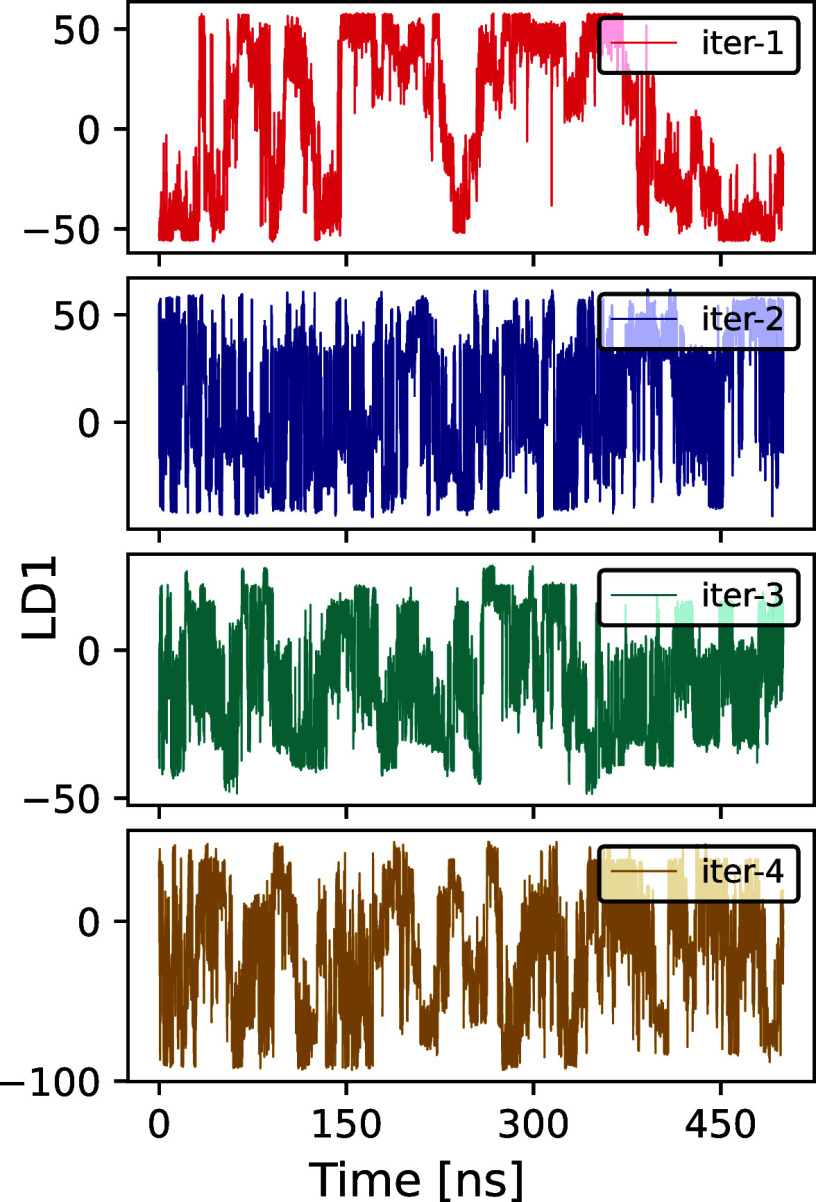
Time course of the biased LDA coordinate for (Aib)_9_. 500 ns of data were used for clustering and training of the next LD coordinate.

To perform iterations, we scan over possible numbers of clusters and compute the log-likelihood for each clustering, as shown in Figure S1. These data were used to pick the “best” number of clusters, as described in ref 26. Figure S2 displays the calculated Bhattacharyya distances for all newly identified clusters relative to the initial states for the last three iterations (see Appendix A for details). The two states nearest to either the left or right side are selected to construct a newly weighted LD1 coordinate for subsequent iterations. The magnitudes of particle displacement vectors acting on individual atoms for all LD1 coordinates are depicted in Figure S3.

From these data, we are also able to compute FE profiles along each LD coordinate, as shown in Figure S4. Because the coordinate changes each time, making it harder to compare iterations to each other, we focus on a previously defined helicity coordinate, ζ, which is the sum of the five central ϕ dihedral angles.^15^ Values of ζ ∼ −5 and ζ ∼ +5 correspond to the right- and left-handed helices, respectively. This CV serves as a consistent reference coordinate to track state transitions and assess the convergence of the FES along it (Figure 3).

**Figure 3:**
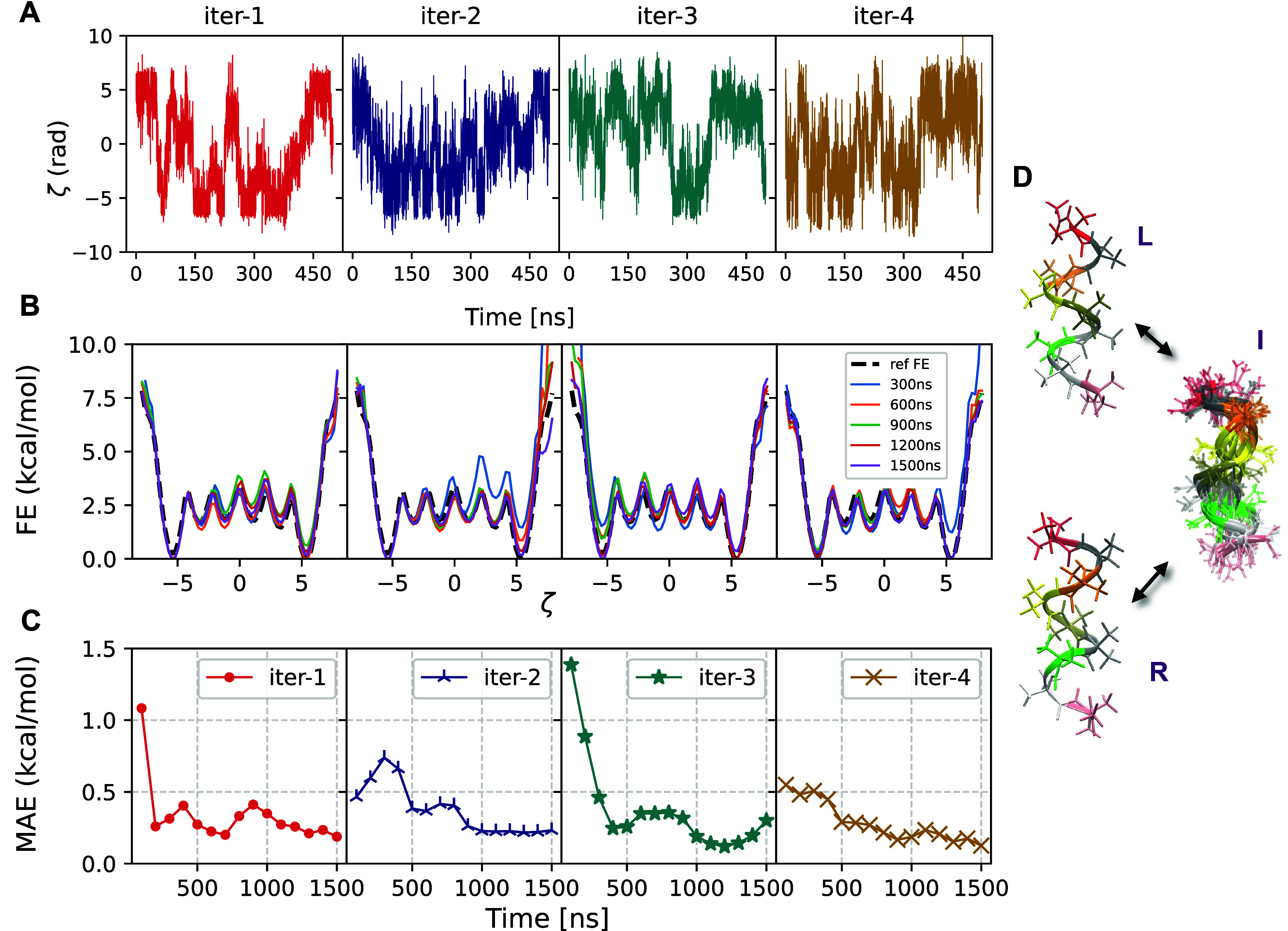
(A) Trajectories for (Aib)_9_ of the physically motivated ζ with time for successive iterations over the time range used to train the next iteration. Each iteration shows transitions between left- and right-handed states, but iteration 4 shows the most transitions per unit time. (B) Free energy (FE) vs ζ for successive iterations, estimated every 300 ns up to time 1.5 μs. The black dashed line is the reference FE, obtained by reweighting from a 4.5 μs long WT-MetaD based on the fourth iteration. (C) Mean absolute error (MAE) computed up to different times from the simulation in each iteration with respect to a reference FE as shown in (B). Only data with FE < 7.5 kcal/mol were used to compute MAE. (D) Representative structures from iteration 1, showing transitions from left (L)- to right (R)-helical states of (Aib)_9_. Intermediate (*I*) shows representative snapshots of configurations having ζ ≈ 0.

Figure 3 illustrates the transitions along ζ during WT-MetaD simulations across all four iterations, along with the corresponding one-dimensional reweighted free energy profiles. The coordinate used for iteration 1 is highly sensitive to the application of bias forces, as previously discussed in ref 9 meaning that gentle biasing had to be applied to prevent “crashing” due to rapid changes in forces. This resulted in a relatively slow sampling of the configurational space. In contrast, here, we observed that the CVs obtained in subsequent iterations are substantially more stable and effective in facilitating extensive sampling of the free energy surface (FES) when employed with higher hill heights and bias factors. For the specific values of the MetaD parameters used, refer to Section 5. Notably, state transitions increase significantly from the second to fourth iteration with consistent MetaD parameters.

To test whether free energy profiles along each coordinate would eventually converge, we extended each simulation to 1.5 μs (Figure S5). Figure S6 displays ζ trajectories over time as well as the final FES for each iteration. In Figure 3B, we show how the estimate of the FES changes across iterations. For all CVs, the final FE profile exhibits left and right states having equal free energy minima within 0.5 kcal/mol. Moreover, we extended our final iteration up to 4.5 μs, which we use as a reference to which we can compare. In Figure 3, we show that the mean absolute error (MAE) relative to this surface decreases rapidly over time in all cases. From this, we observe that the initial coordinate was effective, as reported in our previous work, and subsequent coordinates are more stable but seem to provide approximately similar performance.

As a final experiment on this system, we explored the effect of enforcing equal contributions from the two states when constructing the weighted LD1 coordinate (i.e., assigning each state a combined sample weight of one). This approach was tested for iteration 2 using biased data from the first enhanced sampling simulation. The resulting coordinate performed comparably to the original, as shown in Figure S7.

### 3.2 Performing Iterations on HP35

We previously applied our shapeGMM clustering approach on a 305 μs-long MD trajectory of the fast-folding Nle/Nle mutant of HP35, obtained from the D. E. Shaw Research Group. For our analysis, we selected a six-state representation of the system, which provides an interpretable depiction of the folding and unfolding process. Details of the clustering methodology and cross-validation are discussed in ref 26. The six-state model was trained using 25,000 frames sampled from a data set of approximately 1.5 million frames. In our subsequent study, we demonstrated that a single folding/unfolding coordinate could be derived by performing LDA on frames assigned to the folded and unfolded states from this six-state representation.^9^ Remarkably, this coordinate, trained exclusively on two states, was sufficient to characterize transitions between the folded and unfolded states through physically meaningful configurations. Moreover, it proved to be an effective sampling coordinate when biased in OPES-MetaD simulations.^9^

Here, we implemented the proposed iteration scheme on the system to assess the effectiveness of our approach. The first iteration aligns with the methodology employed in our prior work. Following the procedure outlined in Section 2.1, we conducted a total of three iterations. In the second and third iterations, only 2.5 μs and 1.5 μs of biased data from the previous runs, respectively, were used to train the new wLDA coordinates. The resulting cluster scans for each training iteration are illustrated in Figure S8. For the second iteration, the training process utilized 44,000 samples, with an additional ∼5000 samples reserved for cross-validation. In the final iteration, the data set was expanded to 90,000 training samples, supplemented by 10,000 samples for cross-validation. In each iteration, we computed the Bhattacharyya distance between the newly generated clusters and our predefined folded and unfolded states (see Appendix A). The resulting *D*_B_ data, presented in Figure S9, quantifies the similarity between the two clusters.

The clusters most closely resembling either the folded or unfolded state are selected. The weighted LDA coordinates derived between the new states at each iteration differ from one another, and the coefficients of the LD1 vectors are illustrated in Figure S10. The variation of the LD1 coordinates from OPES-MetaD simulations, performed for every iteration, and the corresponding free energy (FE) profiles computed along them are displayed in Figure S11. Additionally, Figure S12 shows the reweighted 2D free energy surface (FES) projected onto the RMSD space, calculated using those biased simulations.

To assess free energy convergence, each OPES-MetaD simulation was extended, and Figure 4 presents the time trace of LD1 coordinates and 1D FE profiles obtained along with them from the extended simulations for all iterations. While the first simulation ran for approximately 10 μs, we achieved significant convergence to the reference free energy (FE) in the last two iterations within just 3.5 μs. Figure 5 shows the evolution of the MAE in the free energy estimates along the LD1 coordinate in each iteration, computed relative to the reference free energy profile over simulation time. This suggests a notable improvement in the effectiveness of the new coordinates used in iterations 2 and 3 relative to our initial coordinate.

**Figure 4:**
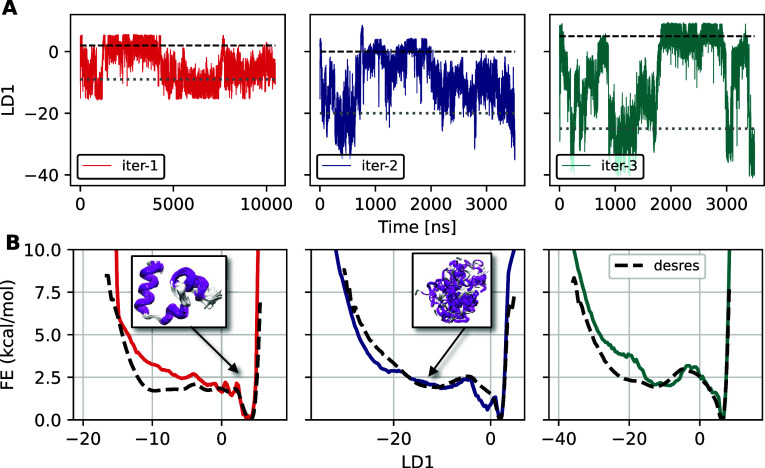
(A) Trajectories of posLDA coordinates with time from extended OPES-MetaD runs for HP35. Horizontal black dashed and gray dotted lines in each case indicate the approximate locations for the folded and unfolded states, respectively. The simulations for iteration 1 ran for 10 μs, and the remaining two ran for approximately 3.5 μs. (B) FE as a function of LD1 for successive iterations. The black dashed line represents the unbiased free energy estimate derived from D. E. Shaw Research data.^43^ The insets highlight representative structures of the folded and unfolded clusters (both taken from iteration 2), illustrating the conformational changes during the transition. Protein conformations are colored according to their secondary structure.

**Figure 5:**
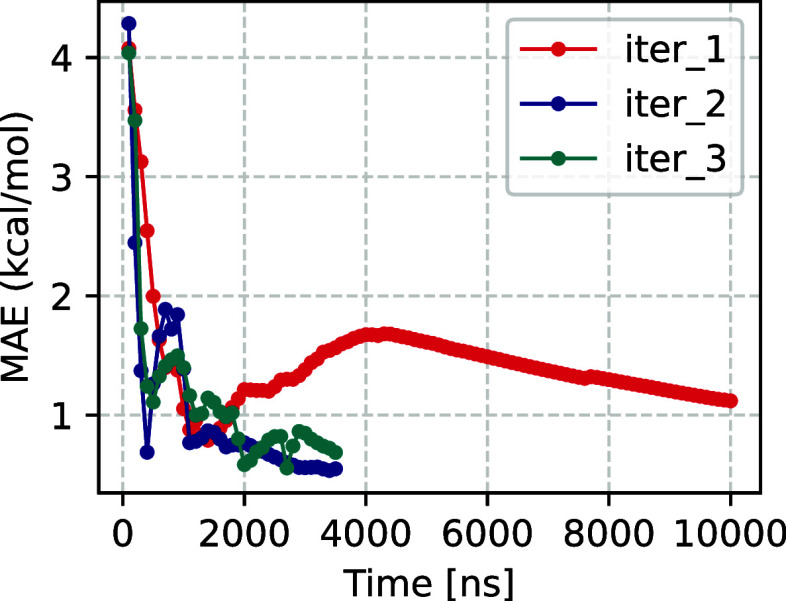
Mean absolute error (MAE) computed up to different times from the simulation in each iteration for HP35, with respect to a reference FE that is obtained from the reference unbiased simulation computed from D. E. Shaw Research data (desres).^43^ Only data with FE < 7.5 kcal/mol are used to calculate the error. Both iterations 2 and 3 show significant improvement over our starting coordinate.

To further evaluate the sampling obtained within these simulations, we computed the 2D free energy surfaces (FES) projected along two RMSD coordinates relative to Helices 1 and 3 for each iteration, as shown in Figure 6, with a reference FES computed from D. E. Shaw Research data depicted as overlaid contour lines. The results clearly demonstrate that the two new iterated wLDA coordinates served as more efficient sampling coordinates, which can sample partially folded structures along the *x*- and *y*-axes more accurately than our initial coordinate. The coordinate in iteration 2 also accurately captures the metastable unfolded bin in the upper right of the landscape, whereas this is still somewhat under-sampled in iteration 3. In Figure S13, we also show how this surface converges versus time, and while both iterations 2 and 3 converge much more quickly than iteration 1, it does suggest that the coordinate in iteration 2 is more efficient, at least when using these OPES-MetaD parameters and assuming the underlying reference surface is converged.

**Figure 6:**
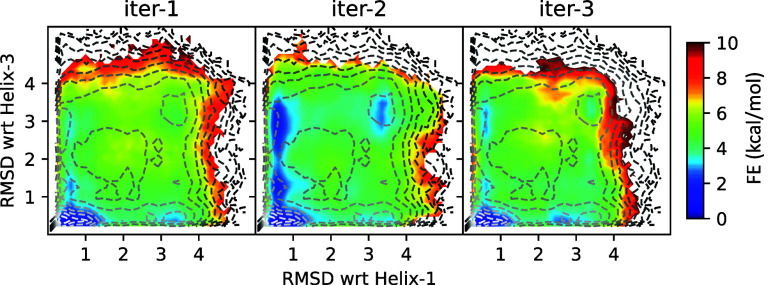
Comparison of 2D FES from three iterations projected along the RMSD with respect to Helices 1 and 3. The color scale represents free energy in kcal/mol. Contour lines indicate the reference free energy estimate derived from D. E. Shaw Research data.^43^ The FESs from iterations 2 and 3 demonstrate improved sampling, capturing a broader region of the free energy landscape with better agreement to the reference.

## 4 Conclusions and Outlook

Our results demonstrate that LDA coordinates based on atomic positions can be iteratively improved using data generated by metadynamics-like sampling. Iteration improves both the stability and sampling efficiency of the resulting CV. We note that these two effects are coupled in that improved stability of the CV also allows us to apply bias more quickly, which improves sampling efficiency. Yet, in our studies, we also find this is not the only effect, and the CV also allows better exploration even when using a similarly gentle MetaD bias to that from earlier iterations.

We speculate that this results from a better estimate of the positional covariance matrix around each metastable state, which produces a more smooth transition between the two targeted states; however, this requires further investigation.

Although our results are promising, some challenges and questions remain. One question in any such approach is: how long should each stage of the iteration be? If a stage of the iteration were run exhaustively to convergence, then there would be no need to iterate to produce a more efficient coordinate. Our results show, for these examples, that it is possible to improve the coordinate by using enough sampling time to have one to two round-trip visits to each state. However, it remains to be investigated whether that generalizes to more challenging systems and whether it produces better results than running using the first CV for as long as all of the iterations combined. We believe that there is an actual improvement in CV quality that results in better estimates, as demonstrated, e.g., by our improvement in convergence rate demonstrated in Figure 5. It also remains an open question as to when to stop the iterations.

Going forward, we will build on this approach for sampling more challenging systems. When going to a larger system, we believe based on our experience that a single linear coordinate will not be sufficient to capture all the slow degrees of freedom when transitioning between two states. We would therefore like to investigate combining the iterative improvement of one posLDA CV that separates the two states of interest with other CVs that promote exploration of other large domain motions. We also would like to investigate cases that include metastable intermediates to see whether a single posLDA CV between the end states is good for sampling or whether we should in fact combine multiple posLDA CVs defined pairwise between metastable states, which would allow us to better sample from, e.g., a starting state to an intermediate state and then from the intermediate state to a final state, via a more physically realistic route.

## 5 Simulation Details

All simulations were performed using GROMACS 2020.4 ^44^ with PLUMED 2.9.0-dev.^25,36^ All analysis scripts, Jupyter notebooks, and PLUMED input files used in the study are available in our paper’s GitHub repository https://github.com/hocky-research-group/Sasmal_posLDA_iteration, on Zenodo 10.5281/zenodo.15530253, and from PLUMED-Nest^25^ on publication.

### 5.1 (Aib)_9_ Simulations

Equilibrated inputs for (Aib)_9_ were provided by the authors of ref 15. In brief, simulations were performed using the CHARMM36m force field and TIP3P water.^45^ MD simulations were performed in NPT with a 2 fs time step at *T* = 400 K. The MetaD parameters used for the first iteration were HEIGHT = 0.005, BIASFACTOR = 2, SIGMA = 0.43, and PACE = 500. For all three remaining iterations, we used HEIGHT = 0.70, BIASFACTOR = 8, SIGMA = 0.55, and PACE = 500, and a multiple time step STRIDE for biasing of 2.^46^ Quadratic upper and lower walls were applied at ∼±10.0 of the maximum and minimum values for each LD1 coordinate, respectively, with a bias coefficient of 125 kcal/mol/Å^2^. Complete details are provided in the PLUMED input files on GitHub.

### 5.2 HP35 Simulations

A 305 μs all-atom simulation of Nle/Nle HP35 at *T* = 360 K from Piana et al.^43^ was analyzed. The simulation was performed by using the Amber ff99SB*-ILDN force field and the TIP3P water model. In that simulation, protein configurations were saved every 200 ps for a total of ∼1.5 M frames. For our simulations, we solvated and equilibrated a fresh system using the same force field at 40 mM NaCl. Minimization and equilibration were performed using a standard protocol http://www.mdtutorials.com/gmx/lysozyme/index.html, at which point NPT simulations were initiated at *T* = 360 K. mdp files for all steps of this procedure and the topology files are available in the GitHub repository of our previous work.^9^ All the OPES-MetaD simulations were performed with γ = 8, Δ*E* = 10 kcal/mol, a pace of 500 steps, and a biasing multiple time step^46^ stride of 2. Quadratic walls were applied for each LD1 coordinate, specific to its range between the upper and lower limits, with a bias coefficient of 125 kcal/mol/Å^2^.

## Supplementary Material



## Appendix A

### Bhattacharyya Distance

The Bhattacharyya distance is a statistical measure used to quantify the similarity between two probability distributions. It is derived from the Bhattacharyya coefficient, which measures the amount of overlap between two distributions. For any two given continuous distributions *p*(*x*) and *q*(*x*), the coefficient is defined as,

A1
BC(p,q)=∫dxp(x)q(x)

and the distance *D*_B_ is given by,

A2
$$D_{B}\left(p,q\right)=-\ln\left(\mathrm{BC}\left(p,q\right)\right)$$

*D*_B_ is a symmetric quantity, which means *D*_B_(*p*, *q*) = *D*_B_(*q*, *p*). It goes to zero for identical distributions and goes to infinity for entirely dissimilar distributions. It is assumed that the compared distributions are well-defined and normalized. If the distributions are too different from each other, the distance can be very large.

If *p*(*x*) and *q*(*x*) are multivariate normal distributions, such as $p\left(x\right)∼N\left(\mu_{p},\Sigma_{p}\right)$ and $q\left(x\right)∼N\left(\mu_{q},\Sigma_{q}\right)$ , then it can be derived to show that *D*_B_(*p*, *q*) is given as,

A3
$$D_{B}\left(p,q\right)=\frac{1}{8}\left[{\left(\mu_{p}-\mu_{q}\right)}^{T}\Sigma^{-1}\left(\mu_{p}-\mu_{q}\right)\right]+\frac{1}{2}\ln\left[\frac{\det\left(\Sigma \right)}{\sqrt{\det\left(\Sigma_{p}\right)\det\left(\Sigma_{q}\right)}}\right]$$

**μ**_*p*_ and **μ**_*q*_ are the mean vectors corresponding to distributions *p*(*x*) and *q*(*x*), with covariances **Σ**_*p*_ and **Σ**_*q*_, respectively. And, $\Sigma =\frac{\Sigma_{p}+\Sigma_{q}}{2}$ is the mean of the two covariances. The first term in eq 17 is the Mahalanobis distance between the two distributions, which quantifies the difference in their locations. The second term accounts for the difference in the shapes of the two distributions and is a measure of the divergence due to differences in their spreads and orientations.

For this work, we implemented the Bhattacharyya distance metric in the similarities module of the shapeGMMTorch package.^24^

## Notes

Training curves for (Aib)_9_ iterations (Figure S1); Bhattacharyya distances for (Aib)_9_ (Figure S2); coefficients of LD coordinates from (Aib)_9_ iterations (Figure S3); FEs vs LD1 for (Aib)_9_ iterations (Figure S4); FEs and time dependence of LD coordinates from (Aib)_9_ 1.5 μs simulations (Figure S5); FEs and time dependence of ζ from (Aib)_9_ 1.5 μs simulations (Figure S6); implementing equal weights for left and right helix (Figure S7); training curves for HP35 iterations (Figure S8); Bhattacharyya distances for HP35 (Figure S9); coefficients of LD coordinates from HP35 iterations (Figure S10); FEs vs LD1 for HP35 iterations (Figure S11); 2D FES on RMSD space from HP35 iterations (Figure S12); and convergence of 2D free energies for HP35 iterations (Figure S13) (PDF)
