# Supplementary material for: Improved Data-Driven Collective Variables for Biased Sampling through Iteration on Biased Data
Source: J Phys Chem B. 2025 Jun 13;129(25):6163–71. doi: 10.1021/acs.jpcb.5c02164 (PMC12207592; doi:10.1021/acs.jpcb.5c02164)
Supplement: Supplementary file 1 [file jp5c02164_si_001.pdf]

Supporting Information for: Improved Data-driven Collective Variables for Biased Sampling through Iteration on Biased Data

Subarna Sasmal,[†] Martin McCullagh,[*,‡] and Glen M. Hocky[*,†]

†*Department of Chemistry and Simons Center for Computational Physical Chemistry, New York University*

‡*Department of Chemistry, Oklahoma State University*

E-mail: martin.mccullagh@okstate.edu; hockyg@nyu.edu

S1 Training curves for $(Aib)_9$ iterations

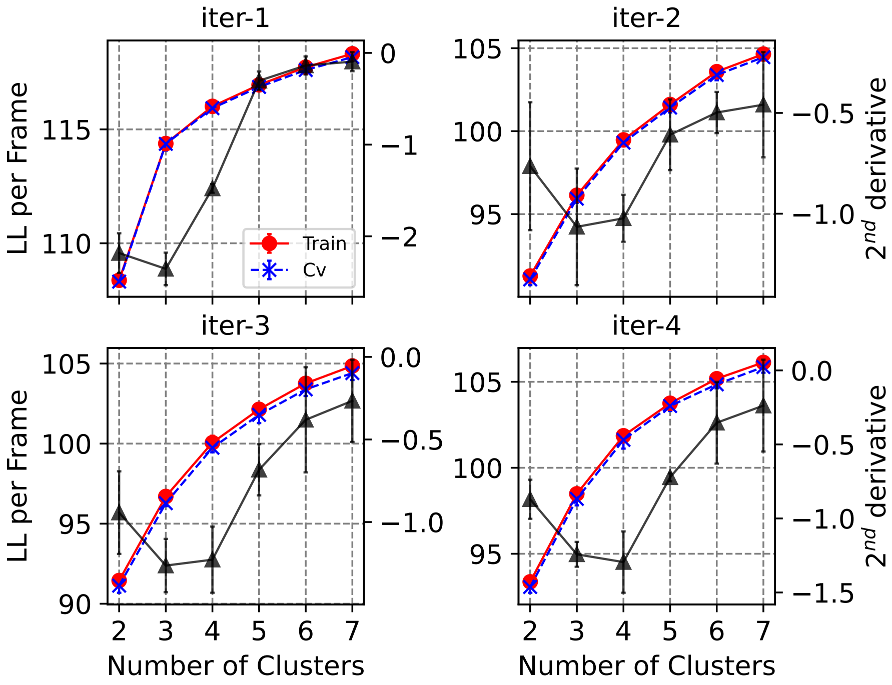

Figure S1: Cluster scans from four successive iterations of $(Aib)_9$. Iteration 2,3 and 4 were performed with data from biased simulations, using 90k training samples and 10k samples for cross validation. First iteration was performed with combined data from two short 20ns long MD simulations initiated form both left and right states. In first iteration, we used 20k frames for training along with 20k for cross validation. Black curves represent 2nd derivatives(with error bars) of log likelihood with respect to number of clusters and minimum value indicates an optimal choice for number of clusters.

S2 Bhattacharyya Distances for $(\text{Aib})_9$

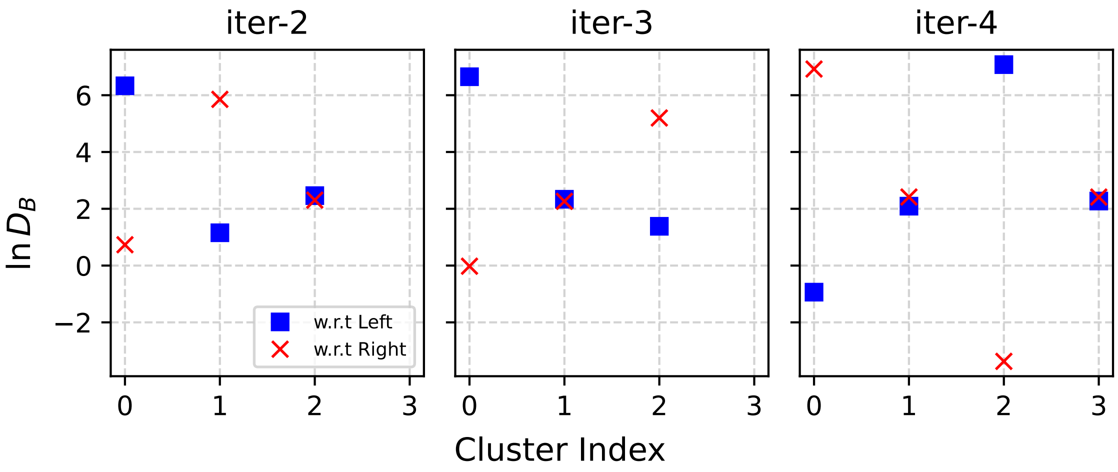

Figure S2: Logarithm of Bhattacharyya distances for all clusters with respect to initial definitions of left and right helical states at every iteration. It gives a measure of similarity between two multivariate normal distributions that represent a cluster. Any two clusters with lower values of $\ln D_B$ are close to each other and those with higher values are far away from each other. It provides a consistent way of defining new left and right states at every iteration in accordance with initial definitions.

S3 Coefficients of LD coordinates from $(Aib)_9$ iterations

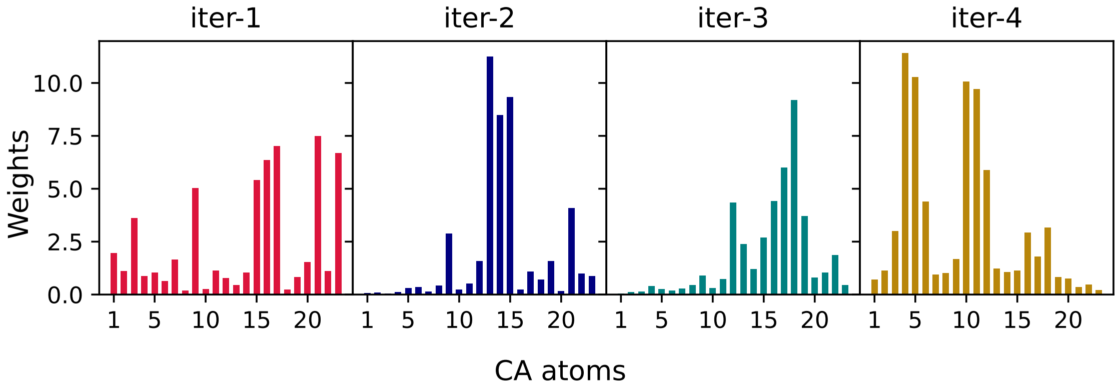

Figure S3: Weights shown are the magnitudes of particle displacement vectors acting on each atom from LD1 after each iteration. In case of $(Aib)_9$, cartesian coordinates of total 23 backbone atoms are used to define LD1 coordinate which is a linear combination of $23 \times 3 = 69$ features with 69 real coefficients. Hence, each particle has a displacement vector of 3 components associated with it. In this figure, it shows the magnitude of those vectors. Weights are considered as contributions of different atoms in making the coordinate. The atoms with larger weights have a larger effect when biasing while those with smaller weights contribute less.

S4 FEs vs. LD1 for $(Aib)_9$ iterations

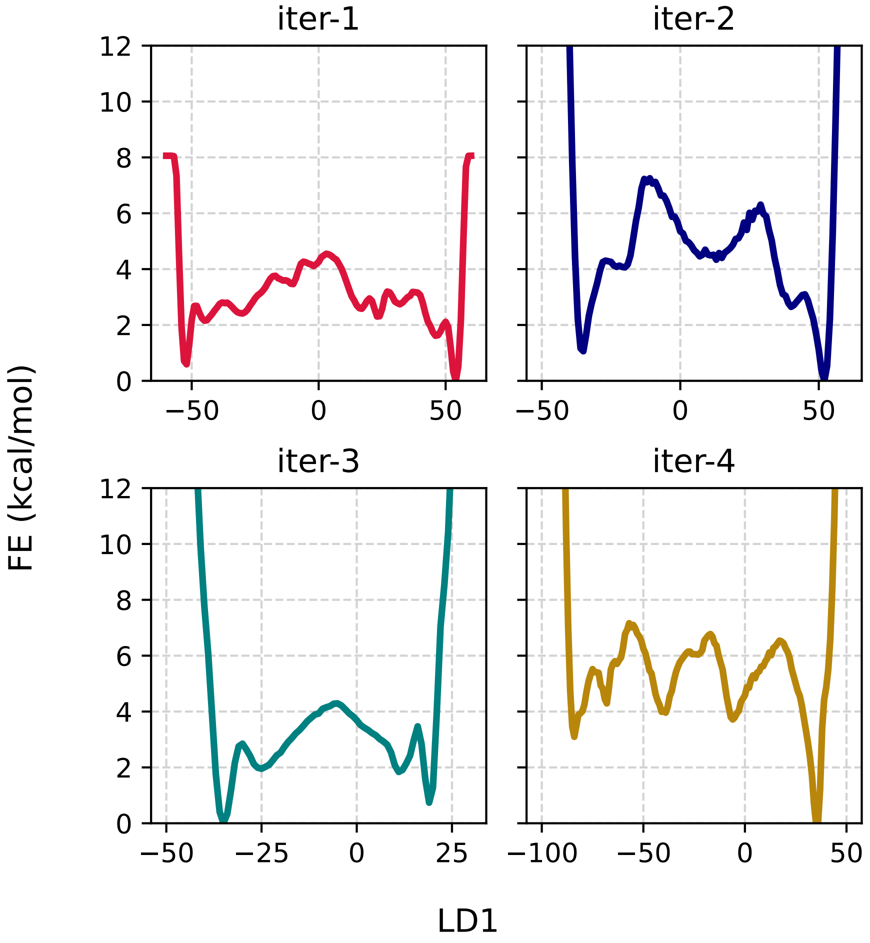

Figure S4: FE profiles along LD1 obtained from 500ns long WT-MetaD simulations in four successive iterations of $(Aib)_9$.

S5 FEs and time dependence of LD coordinates from $(Aib)_9$ 1.5μs simulations

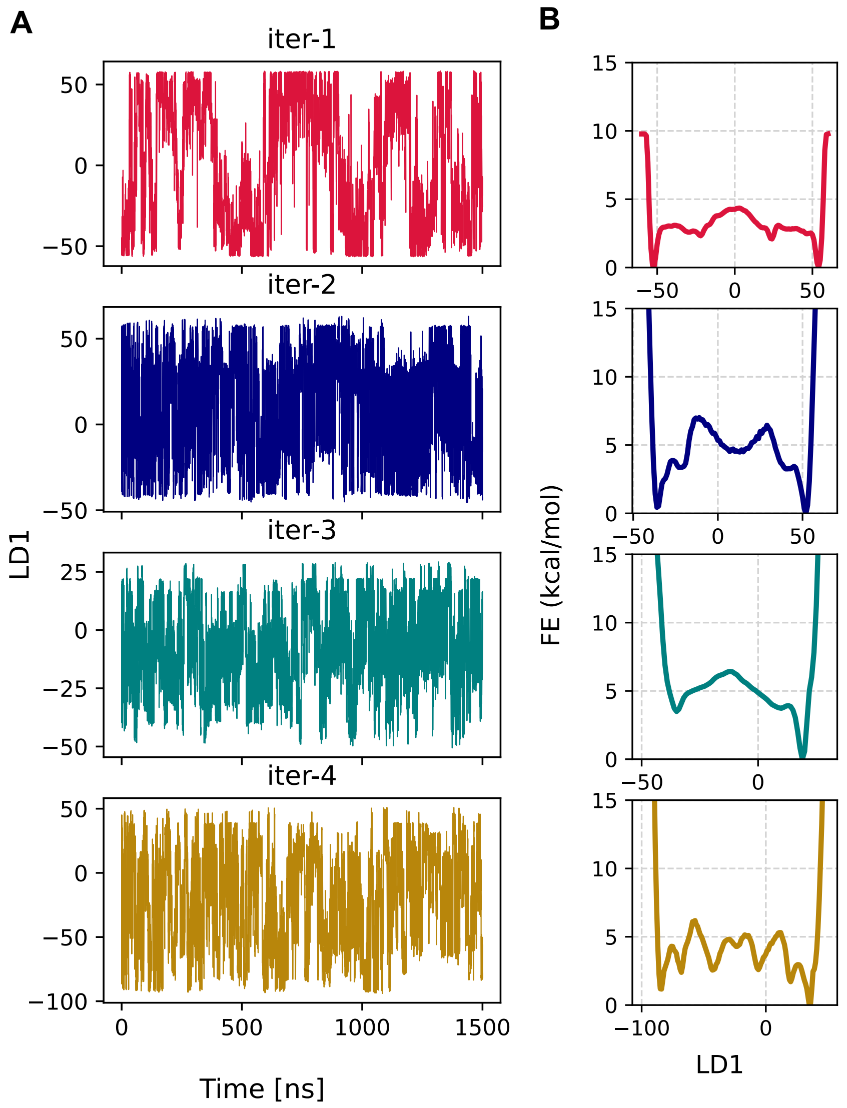

Figure S5: Each WT-MetaD simulation from successive iterations were further extended upto 1.5 μs. (**A**) Fluctuations of LD1 with time and (**B**) FE profiles computed along LD1 in each case by summing all Gaussian hills deposited over the course of the simulations.

S6 FEs and time dependence of ζ from $(\mathrm{Aib})_9$ 1.5μs simulations

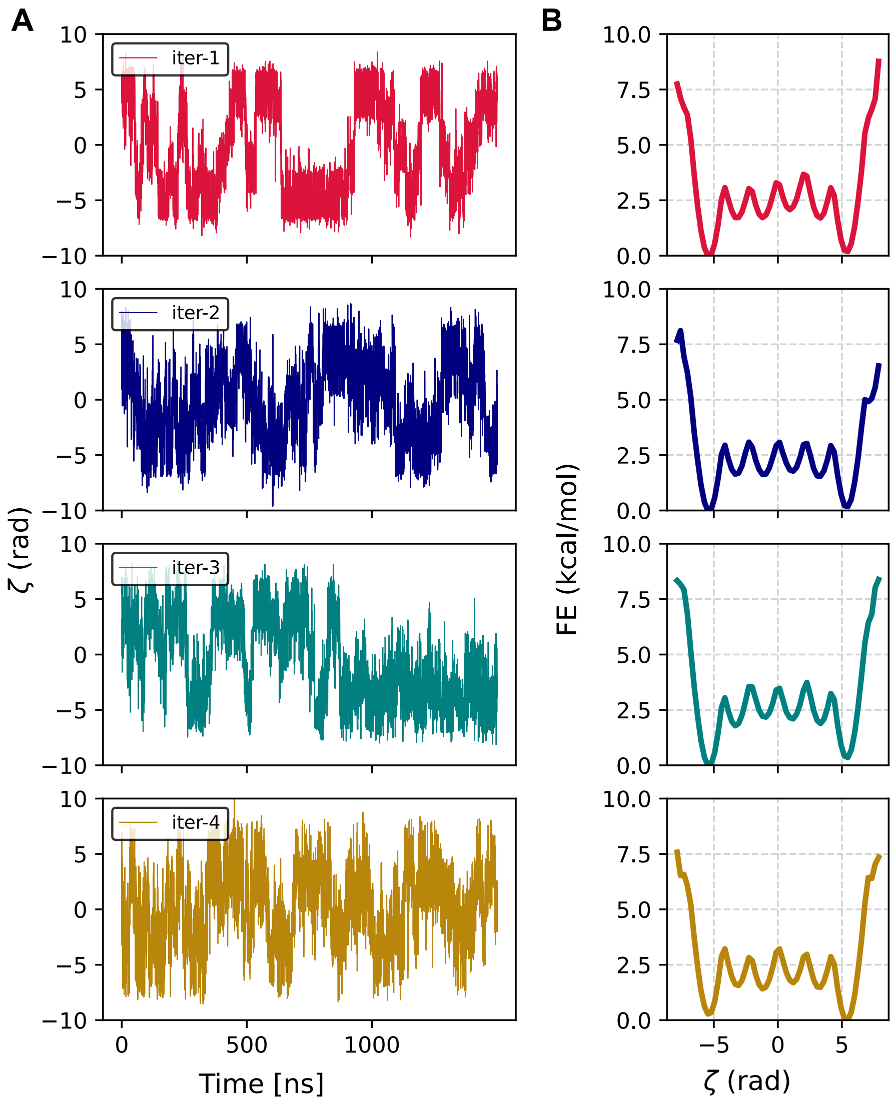

Figure S6: (**A**) fluctuations of ζ with time and (**B**) converged reweighted free energy profiles computed from 1.5μs long WT-Metad simulations. Efficient sampling between left and right states is observed in all cases. All free energy profiles along ζ are converged and symmetric.

S7 Implementing Equal Weights for left and right helix

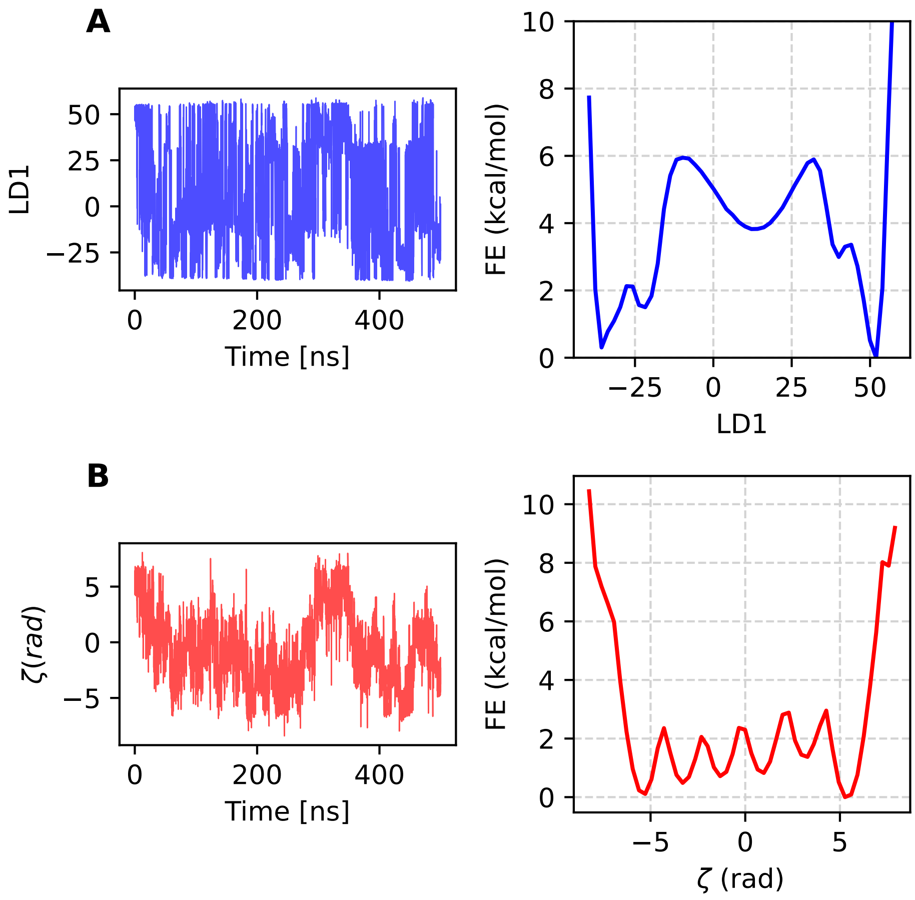

Figure S7: Results from a 500ns long WT-MetaD simulation by biasing LD1 coordinate which is obtained by implementing equal total probability for samples belonging to left and right states. Equal total probability means that sum of weights for all the samples from either left or right state is equal to 1. To test this we used WT-MetaD data from first iteration. After computing the correct weights for samples from biased data, here we normalized the weights separately for left and right states before feeding it into LDA algorithm, so that each state contributes equally to the coordinate. (**A**) LD1 vs. time and free energy profile calculated along LD1 and (**B**) ζ vs. time along with FES along ζ. The MetaD parameters used for this simulation are, HEIGHT=0.01, BF=8, PACE=2000, SIGMA=0.55 and STRIDE=2. Two quadratic walls were applied at LD1=+60.0 and LD1=-60.0 with force constant of 125.0 kcal/mol/Å^2.

S8 Training curves for HP35 iterations

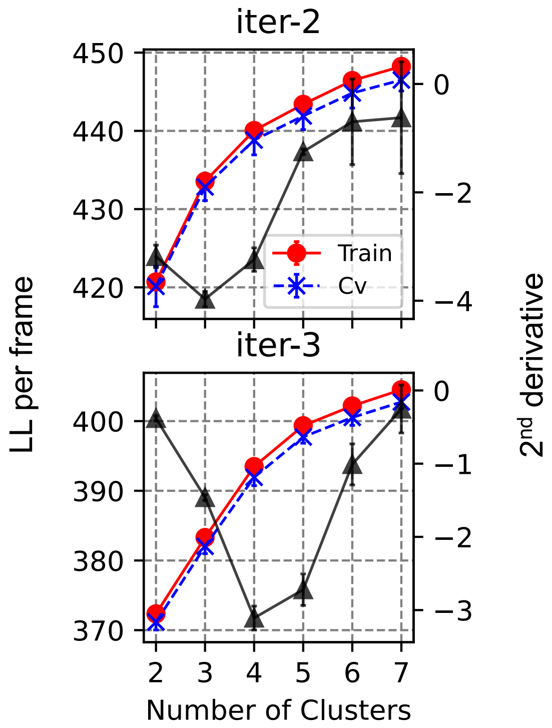

Figure S8: Cluster scans for last two iterations of HP35. The first scan (reported in Ref. 1, not shown here) was performed with 305μs long MD simulation trajectory of HP35 provided by D. E. Shaw Research.[2] The second scan was performed with 2.5μs long OPES-MetaD simulation data with 44k frames for training along with ~5k frames for cross validation. The third scan was performed with 90k samples for training and 10k samples for cross validation, using the biased data from previous 1.5μs long OPES-MetaD simulations. Training curves with error bars are shown in red and cross validations curves with error bars are shown in blue. Black curves represent 2nd derivatives(with error bars) of log likelihood with respect to number of clusters and minimum value indicates an optimal choice for number of clusters.

S9 Bhattacharyya Distances for HP35

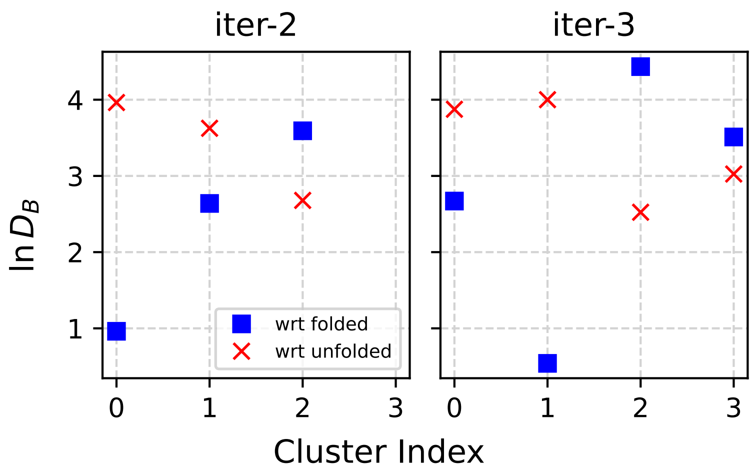

Figure S9: Logarithm of Bhattacharyya distance for all clusters (see Fig. S8) in our HP35 iterations with respect to initial definitions of folded and unfolded clusters.

S10 Coefficients of LD coordinates from HP35 iterations

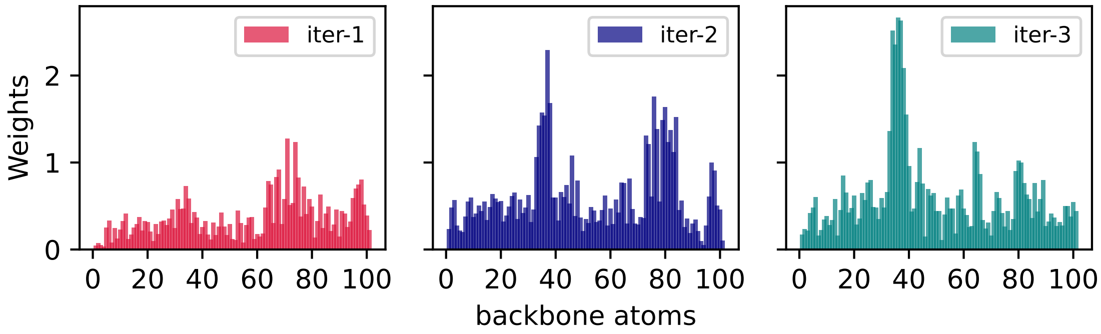

Figure S10: LDA weights at each iteration for HP35. Here, input cartesian coordinates consist of 101 backbone atoms, which is a linear combination of $101 \times 3 = 303$ features with 303 real coefficients.

S11 FEs vs. LD1 for HP35 iterations

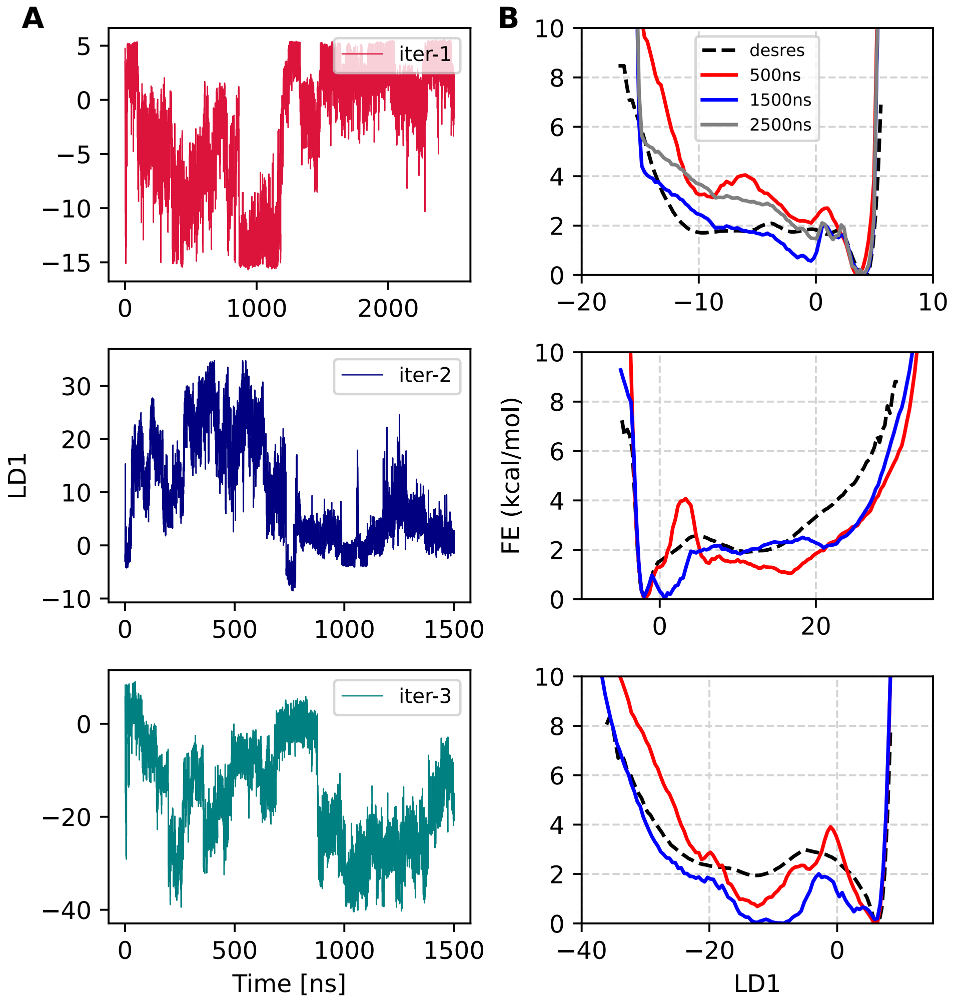

Figure S11: (**A**) Trajectory of LD1 obtained from OPES-MetaD simulations in three successive iterations of HP35. The first simulation is 2.5μs and the remaining two are 1.5μs long. Note that the coordinate obtained at each iteration is different than others. (**B**) FE profiles computed in each oteration. FE profiles calculated using 500ns, 1500ns and 2500ns long data are shown in red, blue, grey colors respectively. In each case for comparison, we also computed a reference FE using 305μs long unbiased MD simulation of villin, provided by D. E. Shaw Research.[2] The reference FE profiles are shown in black dashed lines.

S12 2D FES on RMSD space from HP35 iterations

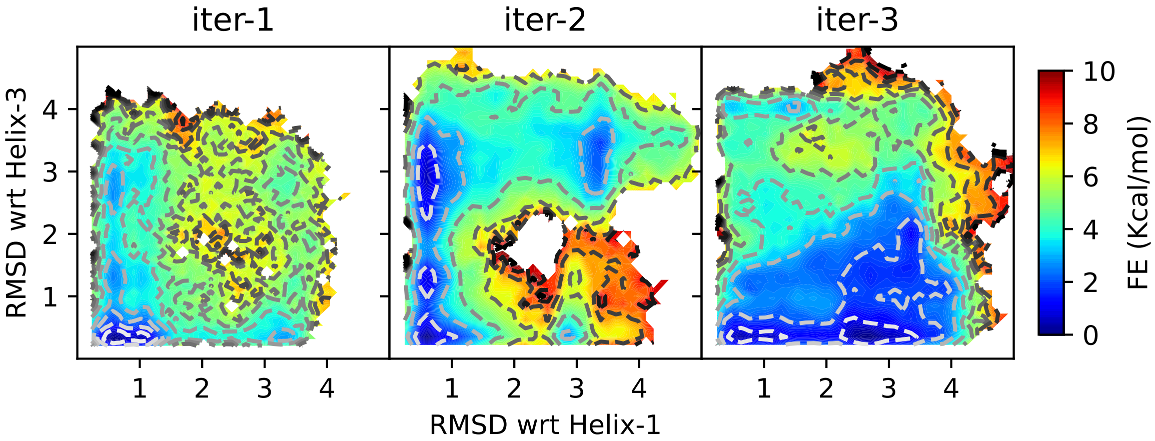

Figure S12: 2D reweighted FES projected along RMSDs (computed using only backbone atoms) with respect to helix-1 and helix-3. To compute the free energy profiles, OPES-MetaD simulation data generated at each iteration is used. The first one is 2.5μs long and the later two are 1.5μs long only (see Fig. S11). This also illustrates the input data which is used in an iteration to generate the next wLDA coordinate.

S13 Convergence of 2D Free Energies for HP35 iterations

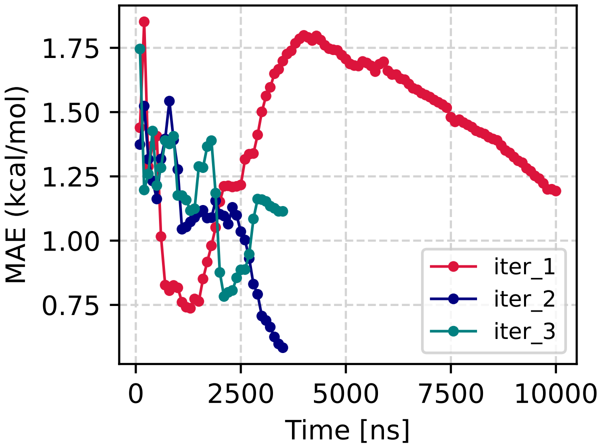

Figure S13: Convergence of 2D FES projected along RMSDs for HP35, showing the mean absolute error (MAE) in kcal/mol across three iterations. The plot illustrates the reduction in error as the simulation progresses, indicating improved convergence of the free energy estimates. Errors are computed with respect to a reference FE computed from long unbiased D.E.Shaw data using a cut-off of 7.5 kcal/mol.

References

(1) Klem, H.; Hocky, G. M.; McCullagh, M. Size-and-Shape Space Gaussian Mixture Models for Structural Clustering of Molecular Dynamics Trajectories. *J. Chem. Theory Comput.* **2022**, *18*, 3218–3230, PMID: 35483073.

(2) Piana, S.; Lindorff-Larsen, K.; Shaw, D. E. Protein folding kinetics and thermodynamics from atomistic simulation. *Proc. Natl. Acad. Sci. U. S. A.* **2012**, *109*, 17845–17850.
